# Reviews

**Published:** 1885-10

**Authors:** 


### REVIEWS.

Lehrbuch der Speciellen Pathologie und Therapie fur Thierarzte. A text-book of Special Pathology and Therapeutics for Veterinarians. By William Dieckerhoff, Professor of Special Pathology and Conductor of the Clinic for Larger Animals at the Royal Veterinary School, Berlin.

Another book with the same title is also advertised by Professor Friedberger, of the Munich Veterinary School, and Fröhner, of the School at Stuttgart.

Neither of these works appear in a completed volume, but in parts. We have received advance sheets of the first work only.

For years the veterinary profession of the world has been very much in need of a text-book upon the special pathology of the domestic animals, of which it could be said that both *scientifically* and *practically*, it was on a par with the best of the modern works upon the same subject in human medicine. The clinical side of most of our books, such as Williams and Robertson, is very well written up, but pathologically they are by no means what they should be in the light of modern research and in comparison with existing conditions in human medicine. Pathologically they consist too much of human patho-anatomy transferred to animal conditions entirely regardless of criticism. Even on this point they are antiquated, being founded more upon Rokitansky than the work of the present Cellular Pathological School. Roell's book that has enjoyed much credit in Europe, never deserved it, and with the exception of the veterinary police, is not equal to either of the English works.

We greet these two new books from fresh authors in this field with the hope and confidence that this want will be filled. Both of them are by noted clinicians. The one before us, by Dieckerhoff will certainly not be a disappointment, the pathology of the diseases so far described being replete in fullness and exactness of description.

It will, also, be an original book in many respects, particularly in regard to the nature of many diseases, especially the pulmonary complications of the horse.

With reference to the so-called "influenza," the author has entered upon the much needed task of analyzing the conditions carefully and endeavoring to show that veterinarians have too frequently been inclined to class quite a number of etiologically different diseases under this one name.

Here we shall find his views decidedly original, and whether we at once agree with them or not, are open to the most earnest consideration.

With regard to influenza proper, which he calls the "Brustseuche" (breast plague), or ,'pneumo-pleuritis contagiosa equorum" typical infectious inflammation of the lungs in the horse. He has opened the question to discussion in a very positive manner as to whether the disease is contagious or infectious in its nature. He looks upon it as a "miasmatic contagious disease." While the whole tenor of his writing is for its contagious nature, still he has so much to say about miasma, stable infection, etc., that one is left in doubt to which he attributes the most essential role in its etiology. The idea that it is contagious has been strongly gaining ground in Germany.

*We do not accept it at all.*

A strictly contagious disease is one in which the original inficiens develope in an animal organism, not outside of it; and only passes from one animal to another, or by means of accidental vehicles. Glanders, the variolæ, rabies. This disease has nothing of that in its nature. Again, we have infections—true malarial diseases, in which the animal must go to a given locality to acquire them—such as fever and ague.

Then we have that somewhat mysterious class of malarial diseases—local in their origin, but in which the animal organism acts as a vehicle through its power of polluting surroundings, but still the diseases are not contagious —cholera, yellow fever, Texas fever, typhoid.

This typhoid-pneumonia of horses most certainly belongs more in this latter class than any other.

That the horse-ail group—strangles, pink-eye, etc., prepares the way for it and frequently exist with it, is a well-known fact. Here we have two distinctly different diseases—two different etiological movements acting upon one and the same organism at the same time.

Changes in the weather causes conditions in horses predisposing them to influenza.

To our mind influenza is a straight miasmatic disease. The ill conditions of stables, manure pits, ventilation, etc., favoring the development of a germ for which the equine organism has some special disposition.

Whether one or more horses in a large stable are attacked by influenza. at approximately the same time. or serially, does not depend upon contagion, but upon the presence of a common cause and outside conditions—usage, exposure and many little things that render such individuals open to infection.

We do not consider these points critically enough. As practitioners we do not often have an opportunity to watch every little indication of indisposition which may act as the artrium for malarial inficientia, which would certainly escape a groom's attention.

Even the fact, quoted by Dieckerhoff, of placing an apparently (?) well horse in a box that had been inhabited by an influenza patient, the disease following is not a convincing proof. We should have the tabulated condition of said "well horse" for a week or more before such a period; should know that the temperature had been normal and even, all this time; that it had never been sluggish or off its feed; that its pulse was and had been regular, and that the mucosæ were normal all the time, before we can accept any such theory. Many horses appear well, work well, yet the next morning, or a few hours, reveals unmistakable indications to the contrary.

We have not made these remarks to be captious, but rather to call attention to one of the strongest places in the book, one which should lead to the most careful study of patients. The above is the result of our own observations, based on the ownership of a large number of horses which we were driving daily, all of which were were kept in a not over clean livery stable in which there were frequent cases of influenza. The next diversion of the author is the description of what he calls "ephemeral pneumonia."

We cannot come to any clear idea of an abortive form of pneumonia, resembling that of influenza in its earliest phenomena, but disappearing in a few days, always ending in recovery, if there is really solidification of any part of the lungs present. The author says "According to my examination the local affection is a serous pneumonia of circumscribed character. The local symptoms, as well as the fever, generally continue every twelve or twenty-four hours; on the next day no signs of disease can be discovered." What a serous pneumonia is, we fail to understand. Such a condition, according to our ideas, would be œdema pulmonum. In fact, we cannot see that the author describes anything more than local congestion, with some dullness of the vesicular respiration. No pneumonia ever closed up in twelve to twenty-four hours from its initiation, that he or any one else ever saw. As no autopsy has ever been made by him of these patients, we beg leave to doubt his conclusions.

It is singular that in this part, which we conclude ends the pulmonary diseases of the horse, that no reference should be made to the ordinary simple, or sporadic pneumonia, which, while frequently characterized by extreme solidification of one lung, rarely extends to both; is never accompanied by gangrenous necrosis, as is the case in influenza, where the fever is never extreme at all in the first few days, when the animal retains, except at this period, a fair appetite; the heart is not much affected in its musculation; where there are seldom, if any, typhoid depression symptoms present; complications are seldom, and recovery generally certain in healthy animals, with nothing but good nursing. There is nothing contagious or malarial infectious about it, but generally follows careless exposure to drafts after work.

This part closes with an exhaustive treatment of the horse-ail—pink-eye, strangle—question. The chapters on anthrax and glanders leave little, if anything, to be desired. What should be done is this: Some competent person should take this work and our two English works, and from them compile a thoroughly good book for English speaking veterinarians. It should be done with a critical consideration of the subject, and in a thoroughly objective manner.

TRICHINIA SPIRALIS AND TRICHINOSIS, INCLUDING AN EXAMINATION OF INDIANA HOGS. By Thomas B. Redding. Prepared under the direction of the State Board of Health of Indiana.

Much as we welcome every attempt at extending our knowledge upon this and kindred questions, we can but think that enough compilatory work has been done with regard to the history, nationality and symptomatology of trichinosis.

This contribution before us is largely a compilation, and inferior to that published by the Army Medical Service by Glazier, in many respects, particularly in the want of illustrations.

It adds nothing new to our knowledge whatever, with the exception of confirming previous reports of the percentage of trichinosis in American hogs: the author having examined 550 hogs—all from Indiana—and found twenty-five of them trichinous—4½ per cent. "The highest percentage of infected hogs were those of the retail butcher shops." We would not be captious, but really cannot see the use, and can readily see the injustice and possible horror. of publishing the names of packing-houses that furnished material. Nor, in criticising this report, would we have it supposed we are seeking cause to find fault with the author.

The point is this. If the hogs of any given State are to be examined, it is not only unjust, but useless to examine a few from one large packing-house or another. That American pork is more highly trichinous than German, is altogether well known at present. What we want to know is: Why is this so?

We must enter upon a critical study of the conditions under which all the hogs in a given State are raised, housed and fattened. We must then examine every hog killed in the State according to their different classes. We shall then know what manner of raising hogs contributes mostly to trichinosis. We shall then, and only then, be able to concentrate our work in a given direction after the cause.

Rats are no more the cause of porcine trichinosis than cats are.

An accidental cause—a dead or sick rat may occasionally be, but the the cause, never. One great failure with our author is that he does not seem to have given any practical study to the subject, with the exception of his few examinations. He makes no attempt to find out how the hogs were raised or where they came from. He does not enter into any critical consideration of the work of others, or consider many of the absurd ideas published in the majority of our government reports, some of which are absolute lies—politically they have another name for this vice.

Still, we welcome even so small a contribution gladly. It is only by piling up the facts that we can eventually hope to make any impression upon the petrified brains of American legislators.

Having begun, we hope the author will extend his labors in the direction of original research, as we have pointed out, instead of extending his work in a larger monograph, for which there is no necessity.

We must warn any one against the idea liable to be gained from reading the article on "Methods, Instruments, etc."

To examine pork for trichinosis is about the easiest thing one can imagine. It does not require any compressors, double knives, etc, simply a pair of curved scissors, two good object glasses, and a microscope with a power of 75 diameters Cut a moderately thin section lengthwise of the muscle with the scissors, put it between your object glassés, press upon them with a rolling motion until the object is transparent, experience will soon show how much, and examine. Soiled and smoked articles require the least soaking of the section in warm water before mounting, simply to soften it. When one is going to enter upon myphological or biological questions, the matter assumes quite another form.

To conclude, we have then a sufficient knowledge o the literature and past history of trichina spiralis. What we need to know now is more biology. Where does the creature come from, and what forms or modes of life has it before the hog swallows it? These given, we can prevent it.

Quarterly Bulletin of the Clinical Society of the New York Post-Graduate Medical School and Hospital. New York: 1885.

We have received the first number of the first volume of a periodical with the above title.

The subject matter, which runs over a hundred pages, is of a high scientific character. Moreover, and this is of more importance, its contents are of much practical value, contributed by experienced physicians, and based on clinical observation.

The article on "Pthisis in relation to Syphilis," by Dr. Porter, is finely illustrated. The price is within the reach of all, $2.00 a year; single numbers being only fifty cents. To be obtained at No. 226 E. Twentieth street.

